# Dual Protection and Dual Methods in Women Living with HIV: The Brazilian Context

**DOI:** 10.1155/2013/540789

**Published:** 2013-06-20

**Authors:** Kiyomi Tsuyuki, Regina María Barbosa, Adriana de Araujo Pinho

**Affiliations:** ^1^Department of Community Health Sciences, UCLA Fielding School of Public Health, 650 Charles E. Young Drive South, 26-070 CHS, Los Angeles, CA 90095-1772, USA; ^2^Population Studies Center (NEPO), University of Campinas (UNICAMP), Cidade Universitaria Zeferino Vaz., Avenida Albert Einstein 1300, Caixa Postal 6166, 13081-970 Campinas, SP, Brazil; ^3^Epidemiology Program, National School of Public Health, Oswaldo Cruz Foundation (FIOCRUZ), Rua Leopoldo Bulhões 1480, 21041-210, Rio de Janeiro, RJ, Brazil

## Abstract

The cooccurrence of HIV and unintended pregnancy has prompted a body of work on dual protection, the simultaneous protection against HIV and unintended pregnancy. This study examines dual protection and dual methods as a risk-reduction strategy for women living with HIV. Data are from a cross-sectional sample of HIV-positive women attended in Specialized STI/AIDS Public Health Service Clinics in 13 municipalities from all five regions of Brazil 2003-2004 (*N* = 834). Descriptive techniques and logistic regression were used to examine dual protection among women living with HIV. We expand the definition of dual protection to include consistent condom use and reversible/irreversible contraceptive methods, we test the dual methods hypothesis that women who use dual methods will use condoms less consistently than women who use only condoms, and we identify predictors of dual protection. Dual protection is common in our sample. Women who use dual methods have lower odds of consistent condom use than women who only use condoms. Among dual method users, we find that women who use an irreversible method use condoms more consistently than women who use a reversible method. Women on ART and with an HIV-serodiscordant partner have greater odds of consistent condom use than their counterparts.

## 1. Introduction

The cooccurrence of HIV and unintended pregnancy has prompted a relatively recent body of work on dual protection, the simultaneous protection against HIV and unintended pregnancy [1]. Dual protection can be achieved through *single method use* (condoms) or *dual method use* (condoms + another contraceptive method). Most studies measure dual protection with condom use, but dual protection definitions must expand to capture condom use consistency and a wider variety of contraceptive methods [1]. Thus far, dual protection studies have focused on samples of women from the general population, have concentrated on developed or African countries, and have not considered HIV-related factors as influential in dual protection behavior. Studies that have considered the benefits of dual protection for people living with HIV show that dual protection can be an effective strategy to prevent HIV transmission to partners and to promote safe childbearing [2–5], which warrants more research in this population. This paper aims to describe dual protection among women living with HIV in Brazil.

Worldwide, HIV is the leading cause of death for women of childbearing age, and up to 64% of all pregnancies are unintended [6, 7]. Brazil has over a third of all people living with HIV in Latin America and the Caribbean [6]. Although the epidemic has become stabilized in Brazil (at 0.5% prevalence) [8], the male to female ratio of new HIV infections fell from 27 to 1 in 1985 to 1.4 to 1 in 2010 [9]. Women represent 35% of the 608,230 people living with HIV/AIDS, and most female infections are in women of childbearing age (83%) and via heterosexual transmission (96%) [9]. Little is known about the dual protection behavior of women living with HIV in Brazil.

Although a substantial proportion of women living with HIV have children after being diagnosed with HIV, women living with HIV in Brazil report lower reproductive intentions than women in the general population [10, 11]. Access to and provision of family planning to these women are essential to enable fertility control and to prevent unintended pregnancy. Although no global estimates exist for unintended pregnancy among women living with HIV, an estimated 30 to 64% of all pregnancies are unintended, with women in South America enduring the greatest burden worldwide [7]. Some studies indicate that the incidence of induced abortion in women living with HIV may be greater than that found in the general population [12]. Other studies find that women living with HIV are often exclusively offered condoms, because they provide dual protection, to the exclusion of being offered a complete basket of contraceptive options [8, 13]. For almost a decade, studies report an unmet need for family planning among women living with HIV in Brazil [12, 14, 15]. Although dual protection is not explicitly promoted in Brazil, most adults in the general population and women living with HIV make clear distinctions between contraceptive methods and the dual protection offered by condoms [16, 17]. 

Given these distinctions it is critical that we better understand dual method use, a much less studied form of dual protection that grants women greater control over their fertility than condom use alone. From a public health perspective, there is resistance to promoting dual methods due to a notion that we call the *dual method hypothesis*, which purports that women who use dual methods for dual protection will use either method or both methods more inconsistently than if they just used one sole method. Several studies provide support for this hypothesis but test it among the general population of women [18, 19]. This study tests the dual method hypothesis and reveals how the dual method hypothesis plays out in a sample of women living with HIV in Brazil.

We also explore how HIV-related factors like antiretroviral therapy (ART) and partner's HIV status affect sexual behavior, condom use, and contraception. People living with HIV may be more sexually active than originally thought. A study in Africa found that, within a three-month period, most people living with HIV (89%) had more than one sexual partner and some (36%) had acquired a new sexual partner [20]. Although the overall effect of ART on sexual behavior and dual protection is relatively unexplored [21], we know that ART lowers viral load, that ART together with condoms helps to prevent HIV transmission to a sexual partner [22], and that ART together with family planning is the most effective way to reduce the vertical transmission of HIV [23]. 

Since 1996, the Brazilian National HIV program has offered universal access to ART and HIV care to people living with HIV. Clinical status and CD4+ count are used to determine when to initiate ART. Generally, ART is initiated when CD4+ count ≤ 500 cells/m^3^ or when the person is symptomatic, coinfected with hepatitis B, at high risk for (or has) cardiovascular disease, being treated for cancer, or pregnant [24]. HAART is offered to all people in HIV-serodiscordant partnerships [24]. Brazil also has protocols in place to prevent vertical transmission, which has resulted in a rapid decline in infants with HIV. Despite the availability of ART, a study in São Paulo, Brazil, finds that less than half of the women living with HIV report using condoms [15]. 

 The effects of partner's HIV status on condom use is mixed depending on the sampled population. Studies find that condom use is typically more consistent among HIV-serodiscordant than seroconcordant couples [21, 25], but one study reports that serodiscordant partners have greater odds of changing sexual partners than seroconcordant partners [20]. Although statistics for Brazil are unavailable, one study reports that serodiscordant partnerships are on the rise and may make up 60% of all relationships [26]. Condom nonuse by HIV-seroconcordant couples may pose a risk for coinfection with other sexually transmitted diseases (STDs) which is linked to decreased CD4+ count and increased viral load [27, 28]. 

This study first describes the characteristics of our sample of women living with HIV in Brazil. Second, we test the dual methods hypothesis that women who use dual methods will do so less consistently than women who use only condoms. Third, we identify important predictors of dual protection in our sample.

## 2. Materials and Methods

Data are from a cross-sectional sample of HIV-positive women attended in Specialized STI/AIDS Public Health Service Clinics in 13 municipalities from all five regions of Brazil between 2003 and 2004 [29]. These clinics provide nearly all ART in Brazil. Women were eligible for the study if they could read and write, were over 18 years, and were HIV positive. A total of 1,777 women completed a self-administered questionnaire and deposited it anonymously into a secure ballot box. The self-administered ballot box technique is associated with fewer refusals, nonresponses, and false responses to sensitive questions and is associated with a greater frequency of reporting socially sanctioned behavior than face-to-face interviews and at times ACASI [30, 31]. The ballot box is a preferred technique to survey sexual behavior in Brazil because it is associated with the confidentiality of political elections, is a validated method in Brazil [31, 32], and is recommended for low-literacy populations (28% of our sample has an elementary school or less education) [33]. 

Our sample includes HIV-positive women who are bi-/heterosexual, of reproductive age, who report at least one sexual partner in the past six months, and with condom use data (*n* = 1, 087). We conducted a sensitivity analysis, removing bisexual women, and results did not differ with the exception that sexual violence became a statistically nonsignificant predictor of consistent condom use. We used listwise deletion to include only cases with complete data. Further, only women who reported using condoms (*n* = 834) were included in our analysis of consistent condom use. We also conducted a missing data analysis and used multiple imputation to ascertain that omitted cases were not significantly different than those included. The research protocol was approved by the IRB of the São Paulo STI/HIV Reference and Training Center. 

The outcome, dual protection, is measured with *consistent condom use*. The consistent condom use measure asked, “*In the last six months, you used condoms: Never, always, or sometimes?”* Respondents who said they *always* used condoms were coded as using consistent condoms, whereas respondents who marked *sometimes* used condoms were coded as using inconsistent condoms. *Sexual and reproductive health *variables include *method of contraception, desire to have children*, *number of live children*, *stable partner*, *history of STD in last six months*, and *lifetime history of sexual violence*. *Method of contraception* comprises three categories (excluding condom use): no method, irreversible method, and reversible method. Irreversible methods include tubal ligation and vasectomized partners. Reversible methods include birth control pills, intrauterine device (IUD), hormonal injection, and diaphragm. The few respondents who use traditional methods were coded as using no method. We classified contraceptive method based on a hierarchy where female-controlled methods took precedence over vasectomy and irreversible methods took precedence over reversible methods. Desire to have children is a binary variable in which the responses “*Yes*” and “*Do not know*” were collapsed into one category. Number of live children includes the number of children a woman had before and after testing positive for HIV. Women were categorized as having a stable partner if they responded positively to having a husband, fiancée, or steady boyfriend. Lifetime history of sexual violence measured any experience of being forced to engage in sexual relations. HIV-related variables include HIV status of sexual partner and currently taking* ART*. We used stepwise logistic regression to select dual protection predictor variables related to sociodemographic characteristics, sexual and reproductive health, fertility, and HIV-related variables.

We employed descriptive and logistic regression techniques to describe dual protection and to identify important predictors of dual protection in our sample. Our first set of analyses uses the chi-square coefficient to assess significant differences related to consistent condom use. Our second set of analyses uses logistic regression to examine the bivariate and adjusted associations between consistent condom use and predictors. Prior to entering each variable into an adjusted model, we conducted a bivariate analysis to assess the association between each independent variable and dual protection. Results for significant predictors of dual protection are displayed in the logistic models, but all independent variables are included in adjusted models. STATA 12 was the software used to conduct all analyses.

## 3. Results and Discussion

### 3.1. Results

Table 1 describes the characteristics of our sample. Women averaged 32 years old and most had a junior high school education or more (73%). Most women either used no method of contraception (51%) or an irreversible method (34%), did not desire to have children (58%), had 2.11 children on average, and were in a stable relationship (87%). The majority of women had no history of STDs in the past six months (81%) and had no history of sexual violence (78%). Most women were in an HIV-serodiscordant relationship (47%) and were taking ART (72%).

Table 2 presents the distribution of consistent condom use by independent variables. Dual protection was prevalent in our sample with most women using condoms consistently (77%). Women who used only condoms for dual protection reported more consistent condom use (82%) than women who used dual methods (condoms + another method) for dual protection (72%). Among dual method users, 74% of the women who use an irreversible method report using condoms consistently versus only 66% of women who use a reversible method. Women not using dual protection (13%) either used no contraceptive method (42%), an irreversible method (37%), or a reversible method (22%). Women who use consistent condoms are slightly older and more educated than women who do not. Women who do not desire to have children report more consistent condom use (80%) than women who desire to have children (74%). Women with no lifetime history of sexual violence report more consistent condom use (79%) than women with a history of sexual violence (71%). Women with an HIV-negative partner report more consistent condom use (86%) than women who do not know their partners' HIV status (71%) and women with HIV-positive partners (68%). Women on ART report more consistent condom use (80%) than women who are not on ART (68%). 

Table 3 presents the bivariate and adjusted models of independent variables on the odds of consistent condom use. Women who use irreversible methods have 0.59 times the odds and women who use reversible methods have 0.48 times the odds, of using consistent condoms than women who use only condoms for dual protection, net of other factors. Women who desire to have children have 0.63 times the odds of using consistent condoms than women who do not desire children, net of other factors. Women with a lifetime history of sexual violence have 0.64 times the odds of using consistent condoms than women without a history, net of other factors. Women with HIV-negative partners have 3.23 times the odds of using consistent condoms than women with HIV-positive partners, net of other factors. Women not on ART have 0.56 times the odds of using consistent condoms than women on ART, net of other factors. 

### 3.2. Discussion

Our study finds a high prevalence of dual protection among women living with HIV. Consistent condom use (77%) was much greater in our sample of women living with HIV than women in the general population at the time (34% in steady relationships and 66% in casual relationships) [34]. One study in Brazil suggests that women increase consistent condom use from prediagnosis (71%) to postdiagnosis (86%) [14]. High levels of condom use in women living with HIV may be the result of the Brazilian government's extensive effort to promote condom use in populations most vulnerable for HIV infection. Despite high levels of condom use, 23% of our sample used no dual protection and 9% of our sample used no dual protection and no form of contraception. The lack of condom and contraception may reflect a desire to conceive [35, 36]. However, a closer look at our data reveals that only 60% of the women who do not use condoms or contraception report fertility desires, highlighting a need for intervention.

Our study provides some support for the dual methods hypothesis and adds a caveat to the hypothesis. The dual methods hypothesis argues that women who use dual methods for dual protection will use either one method or both methods inconsistently; some studies indicate that the more effective the contraceptive method, the more inconsistent the condom use [17, 37]. We find that women who use only condoms use them more consistently than women who use dual methods; this finding is also supported by Whiteman et al. [5]. Other studies that consider dual method use among women living with HIV demonstrate that dual method use is a viable option for dual protection but do not measure condom use consistency [2–4]. Mark et al. [2] finds the adoption of a nonbarrier contraceptive method does not lead to a decline in condom use or an increase in pregnancy. Ngure et al. [3] find that women living with HIV can successfully uptake dual methods with appropriate intervention. However, Heffron et al. [4] use data from a multinational prospective study of HIV-serodiscordant couples in Africa to show modest declines in condom use with the uptake of another contraceptive method. Mixed findings likely result from differences in samples, interventions, and contexts. However, these studies highlight the promise of promoting dual methods for dual protection in this population.

Our study elaborates on the dual methods hypothesis. Our findings are contrary to the belief that the more effective the contraceptive method, the more inconsistent the condom use [17]. We find that, among dual method users, women who use an irreversible method use condoms more consistently than women who use a reversible method. A possible explanation for this finding may be the triple burden faced by women living with HIV where, on a daily basis, women must manage ART protocols, prevent HIV transmission, and prevent unintended pregnancy. A closer look at our data reveals a pattern in which condom use consistency is greatest among women who use a long-term contraceptive method (sterilization, IUD, and hormonal injection) followed by women who use contraceptive methods that require daily attention (contraceptive pill). Female sterilization may serve to alleviate women from the daily burden of contraception, allowing them to focus on consistent condom use. Moreover, for women on ART, taking the oral contraceptive pill adds yet another pill to their daily medication regimens, could cause undesirable side-effects, and could interact with ART to become a less effective method of contraception [38]. In light of these factors, future research should consider how consistency of condom use, consistency of reversible contraceptive use, and ART adherence interact; an analysis is not permitted with our data. 

We find dual protection is determined by partner's HIV status. There is a growing body of literature that links the ability of women living with HIV to use condoms and contraception to a positive relationship context (i.e., quality of relationship, partner dynamics, and communication) [39–41]. Our study finds that women with HIV-serodiscordant partners report the most consistent condom use, followed (in order) by women who do not know their partners' HIV status, and women with HIV-seroconcordant partners. Our findings are supported by other studies that find the most consistent condom use is among HIV-serodiscordant couples [21, 25]. Women in HIV-serodiscordant relationships are likely to use more consistent condoms because they have disclosed their HIV status to their partners and are in “known” discordant relationships. These women are therefore clearer with their partners about the risks involved in having unprotected sex and more compelled to protect the health of their partners [42–44]. It is also possible that women who do not know their partners' status are likely to be in less committed relationships and, therefore, are not compelled to disclose their HIV-positive status to their sexual partner, are less able to negotiate condom use, and are not as concerned for their sexual partners' health to use consistent condoms [42, 44–46]. 

Women with HIV-seroconcordant couples had the lowest consistent condom use in our study, with over 30% not using consistent condoms. Relationship factors, like communication between HIV-seroconcordant partners, may lead to decisions to discontinue condoms due to the lack of perceived risk and the desire for sensation and intimacy [39, 47]. However, unprotected sex among HIV-seroconcordant partners could expose women to coinfection with other STDs, which has been linked to decreases in CD4+ count and increases in HIV viral load [27, 28]. In addition, condom nonuse in HIV-seroconcordant couples may put them at risk of reinfection with other variants of HIV, including drug-resistant viral strains, which could hasten seroconversion to AIDS and potentially complicate ART protocols [48]. Although this phenomenon is not well understood, the potential repercussions are especially disconcerting for a context like Brazil, where ART is freely provided by the government. In fact, there is so much growing concern of the emergence and transmission of antiretroviral drug resistance (ADR) in Brazil that the Ministry of Health has started to monitor ADR in the most populated state capitals. Several studies have reported the prevalence of ADR in Brazil to vary from 1.4 to 12.7% [49–51], and one study in a national sample of MSM found the prevalence to be as high as 21–36% [52]. 

ART is positively related to consistent condom use in our sample, a finding supported by other studies [21, 53, 54]. Data from the Swiss HIV Cohort Study indicate that people living with HIV who are on ART have lower odds of engaging in unsafe sex than those who are not on ART [21]. This finding is also supported by several literature reviews [53, 54]. ART requires frequent visits to healthcare centers which may be linked to greater exposure to safe sex counseling, greater access to healthcare and to condoms, and health care seeking behavior. Other studies find that safer sexual behavior is related to ART adherence [2, 42] and that some people living with HIV believe condom use is essential for ART to be effective [55]. Evidence suggests that those who care for themselves by adhering to ART regimens may have greater odds of also protecting their sexual partners by using condoms consistently. On the contrary, we find that women who are not on ART have lower odds of consistent condom use. Condom nonuse may occur in women who are not on ART because they feel healthy and do not perceive the need to use, or cannot negotiate, condoms. Lack of condom use may also result from a limited knowledge of HIV transmission and disease dynamics and insufficient exposure to condom messaging. In any case, women living with HIV who are not on ART are also not likely to be connected with HIV services and may not know their HIV viral load, the most important determinant of HIV transmission risk in HIV-serodiscordant partners [56]. This finding highlights the need to counsel women, upon HIV-positive diagnosis, about transmission risk and to address barriers to condom use.

Several limitations of this study should be considered. First, data are from a convenient sample of women living with HIV in Brazil. Although this limits the generalizability of our findings, the Specialized STI/AIDS Health Service Clinics provide care to most HIV cases in each municipal; there is no reason to assume our sample is different than the greater population. Second, data are from a cross-sectional survey and employ questions that require the respondent to recall sexual behavior. This caveat may have introduced a recall bias that could have led the respondent to inaccurately recall information. However, the secret ballot box technique used for survey collection has been associated with a more accurate response rate to sensitive questions regarding sexual behavior than other modes of survey administration [30–33]. 

## 4. Conclusions

Our study has three main findings. First, dual protection is a common practice among our sample of women living with HIV in Brazil. Second, we test the dual methods hypothesis and find that women who use dual methods have lower odds of consistent condom use than women who use a single method (condoms). Of dual method users, we find women who use irreversible methods use consistent condoms more than women who use reversible methods. Third, we find that women who are on ART and who have HIV-serodiscordant partners have greater odds of consistent condom use than their counterparts. This study highlights dual method use as a promising form risk-reduction strategy for women living with HIV.

## Figures and Tables

**Table 1:** Sociodemographic, sexual and reproductive, and HIV-related characteristics of women living with HIV/AIDS of reproductive age in Brazil, 2003-2004.

Total *N* (%)	834 (100%)^†^
SOCIODEMOGRAPHIC VARIABLES	
Age (mean (std. dev))	32.0 (6.86)
Education	
Elementary school or less	230 (28%)
Junior high school	308 (37%)
Some high school or more	296 (36%)
SEXUAL AND REPRODUCTIVE VARIABLES	
Method of contraception	
No method	424 (51%)
Irreversible method	285 (34%)
Reversible method	125 (15%)
Desire to have children	
No	483 (58%)
Yes/don't know	351 (42%)
Number of live children (mean (std. dev))	2.11 (1.63)
Stable partner	
No	113 (14%)
Yes	721 (87%)
History STD in last 6 months	
No	675 (81%)
Yes	159 (19%)
Lifetime history sexual violence	
No	647 (78%)
Yes	187 (22%)
HIV-RELATED VARIABLES	
HIV status of partner	
HIV positive	324 (39%)
Does not know	119 (14%)
HIV negative	391 (47%)
Woman on ART	
Yes	603 (72%)
No	231 (28%)

STD: sexually transmitted disease.

ART: antiretroviral therapy.

^†^Column percentages may not sum to 100% due to rounding.

**Table 2:** Distribution of sociodemographic, sexual and reproductive, and HIV-related variables by consistent condom use among women living with HIV/AIDS of reproductive age in Brazil, 2003-2004.

	Consistent condom use	
	(*N* = 834)	
	No	Yes

Total *N* (%)	192 (23%)	642 (77%)

SOCIODEMOGRAPHIC VARIABLES		
Age (mean (std. dev))	*P* = 0.020	
	31.1 (6.94)	32.2 (6.83)
Education	*P* = 0.020	
Elementary school or less	58 (25%)	172 (75%)
Junior high school	82 (27%)	226 (73%)
Some high school or more	52 (18%)	244 (82%)
SEXUAL AND REPRODUCTIVE VARIABLES		
Method of contraception	*P* = 0.001	
No method	77 (18%)	347 (82%)
Irreversible method	73 (26%)	212 (74%)
Reversible method	42 (34%)	83 (66%)
Desire to have children	*P* = 0.042	
No	99 (21%)	384 (80%)
Yes/don't know	93 (27%)	258 (74%)
Number of live children (mean (std. dev))	*P* = 0.544	
	2.17 (1.71)	2.09 (1.55)
Stable partner	*P* = 0.148	
No	20 (18%)	93 (82%)
Yes	172 (24%)	549 (76%)
History STD in last 6 months	*P* = 0.357	
No	151 (22%)	524 (78%)
Yes	41 (26%)	118 (74%)
Lifetime history sexual violence	*P* = 0.031	
No	138 (21%)	509 (79%)
Yes	54 (29%)	133 (71%)
HIV-RELATED VARIABLES		
HIV status of partner	*P* < 0.001	
HIV positive	103 (32%)	221 (68%)
Does not know	35 (29%)	84 (71%)
HIV negative	54 (14%)	337 (86%)
Woman on ART	*P* < 0.001	
Yes	119 (20%)	484 (80%)
No	73 (32%)	158 (68%)

STD: sexually transmitted disease.

ART: antiretroviral therapy.

**Table 3:** Binomial logistic regression estimates of sexual and reproductive and HIV-related variables on the odds of consistent condom use among women living with HIV/AIDS of reproductive age in Brazil, 2003-2004^†^.

	Consistent condom use (*N* = 834)	
	Bivariate model	Adjusted model
	OR (CI)	OR (CI)

SEXUAL AND REPRODUCTIVE VARIABLES		
Method of contraception		
No method	ref.	ref.
Irreversible method	0.64	0.59
	(0.45, 0.93)	(0.39, 0.90)
	*P* = 0.018	*P* = 0.013
Reversible method	0.44	0.48
	(0.28, 0.68)	(0.30, 0.77)
	*P* < 0.000	*P* = 0.002
Desire to have children		
No	ref.	ref.
Yes/don't know	0.72	0.63
	(0.52, 0.99)	(0.44, 0.92)
	*P* = 0.043	*P* = 0.018
Lifetime history sexual violence		
No	ref.	ref.
Yes	0.67	0.64
	(0.46, 0.96)	(0.43, 0.96)
	*P* = 0.031	*P* = 0.030
HIV-RELATED VARIABLES		
HIV status of partner		
HIV positive	ref.	ref.
Does not know	1.12	1.30
	(0.71, 1.77)	(0.81, 2.11)
	*P* = 0.632	*P* = 0.282
HIV negative	2.91	3.23
	(2.01, 4.21)	(2.08, 5.02)
	*P* < 0.000	*P* < 0.000
Woman on ART		
Yes	ref.	ref.
No	0.53	0.56
	(0.38, 0.75)	(0.39, 0.81)
	*P* < 0.000	*P* = 0.002

Logistic regression; OR: odds ratio; CI = 95% confidence interval.

^†^Adjusted models are controlled for by number of children, stable partner, history of STD in last 6 months, age, and education.

ART: antiretroviral therapy.
